# MaDoPO: Magnetic Detection of Positions and Orientations of Segmented Deep Brain Stimulation Electrodes: A Radiation-Free Method Based on Magnetoencephalography

**DOI:** 10.3390/brainsci12010086

**Published:** 2022-01-08

**Authors:** Mevlüt Yalaz, Nicholas Maling, Günther Deuschl, León M. Juárez-Paz, Markus Butz, Alfons Schnitzler, Ann-Kristin Helmers, Michael Höft

**Affiliations:** 1 Microwave Engineering, Christian-Albrechts-Universität zu Kiel, 24143 Kiel, Germany; mh@tf.uni-kiel.de; 2 Boston Scientific Corporation, Santa Clarita, CA 91355, USA; Nicholas.Maling@bsci.com (N.M.); Leon.JuarezPaz@bsci.com (L.M.J.-P.); 3 Department of Neurology, Christian-Albrechts-Universität zu Kiel, 24105 Kiel, Germany; g.deuschl@neurologie.uni-kiel.de; 4 Institute of Clinical Neuroscience and Medical Psychology, Medical Faculty, Heinrich-Heine-Universität Düsseldorf, 40225 Düsseldorf, Germany; butzm@hhu.de (M.B.); alfons.schnitzler@hhu.de (A.S.); 5 Department of Neurosurgery, Christian-Albrechts-Universität zu Kiel, 24105 Kiel, Germany; Ann-Kristin.Helmers@uksh.de

**Keywords:** deep brain stimulation, magnetoencephalography, segmented DBS electrode, bipolar electrode configuration, localization, rotational orientation detection

## Abstract

Background: Current approaches to detect the positions and orientations of directional deep brain stimulation (DBS) electrodes rely on radiative imaging data. In this study, we aim to present an improved version of a radiation-free method for magnetic detection of the position and the orientation (MaDoPO) of directional electrodes based on a series of magnetoencephalography (MEG) measurements and a possible future solution for optimized results using emerging on-scalp MEG systems. Methods: A directional DBS system was positioned into a realistic head–torso phantom and placed in the MEG scanner. A total of 24 measurements of 180 s each were performed with different predefined electrode configurations. Finite element modeling and model fitting were used to determine the position and orientation of the electrode in the phantom. Related measurements were fitted simultaneously, constraining solutions to the a priori known geometry of the electrode. Results were compared with the results of the high-quality CT imaging of the phantom. Results: The accuracy in electrode localization and orientation detection depended on the number of combined measurements. The localization error was minimized to 2.02 mm by considering six measurements with different non-directional bipolar electrode configurations. Another six measurements with directional bipolar stimulations minimized the orientation error to 4°. These values are mainly limited due to the spatial resolution of the MEG. Moreover, accuracies were investigated as a function of measurement time, number of sensors, and measurement direction of the sensors in order to define an optimized MEG device for this application. Conclusion: Although MEG introduces inaccuracies in the detection of the position and orientation of the electrode, these can be accepted when evaluating the benefits of a radiation-free method. Inaccuracies can be further reduced by the use of on-scalp MEG sensor arrays, which may find their way into clinics in the foreseeable future.

## 1. Introduction

Deep brain stimulation (DBS) is a neurosurgical procedure in which electrodes are placed within the brain to electrically stimulate specific target areas, thereby modulating dysregulated neural circuits [1]. Due to the relative safety, therapeutic efficacy, and post-surgically modifiable nature of DBS, it has become a common surgical procedure over the past three decades. To date, it is estimated that over 200,000 patients have been implanted in over 700 centers worldwide [2,3]. While DBS is most commonly used to treat movement disorders, such as Parkinson’s disease and essential tremor, it is increasingly being investigated for its therapeutic potential regarding a range of other brain diseases, including conditions such as neuropathic pain and epilepsy. Estimating the volume of tissue activated (VTA) has demonstrated clinical advantages in programming efficacy [4] and provides a method for optimizing stimulation while avoiding side effects. Conventional DBS electrodes, which have a linear array of ring contacts, offer limited control over an approximately spherical VTA. Directional electrodes with contacts divided into three segments along the circumference of the electrode offer the possibility to steer the VTA inside the target in a desired stimulation direction to prevent the current from spreading to adjacent fibers or nuclei [5,6]. Clinical studies have reported improved therapeutic effects and lower side effect thresholds for directional DBS [7,8]. To realize the full potential of this directional stimulation technology, the correct position and orientation of the electrode in the patient-specific neuroanatomy must be precisely determined in order to meaningfully interpret the observed stimulation’s effects on the individual patient and to guide and facilitate neurostimulator programming.

Current approaches to electrode localization rely on the fusion of pre- and postoperative neuroimaging data. All images are subject to metal artifacts, which increase localization errors in poor quality images. In addition, significant discrepancies between electrode centers estimated by computed tomography (CT) and magnetic resonance imaging (MRI) have been reported, and differences in localization results between different widely used software programs have been noted [9,10,11]. Furthermore, CT exposes patients to ionizing radiation, whereas MRI cannot always be performed for safety reasons. Similar limitations also exist in electrode orientation detection. Current methods for determining electrode orientation include anteroposterior and lateral X-rays, standard CT, flat-panel CT, and rotational fluoroscopy [12,13,14]. Techniques using X-rays and CT rely on high-quality images because specific artifact patterns generated by the radiopaque orientation markers, which are located dorsal to the electrode contacts, must be calculated from these images. However, the underlying symmetric artifact has 180° symmetry, which limits orientation detection to two possible solutions. Neither imaging technique is currently capable of resolving this ambiguity without further measurements. Recently, solutions to the ambiguity of artifact symmetry have been proposed, but additional X-ray images are required to resolve 100% of cases [15,16]. Rotational 3D fluoroscopy seems to be a promising alternative [12], but all existing methods for orientation detection expose patients to ionizing radiation, which may be detrimental to patients. In addition, electrode migration, electrode torsion, and any trauma or surgical intervention may cause undesired electrode displacement and rotation, requiring the patient to be re-exposed to radiation or X-ray imaging. This would hinder further research into whether and to what extent directional electrodes move and continue to rotate after implantation, and long-term clinical studies may therefore be very difficult to conduct.

Alternative imaging techniques, such as electroencephalography (EEG) and magnetoencephalography (MEG), provide alternative imaging modalities for determining electrode positions and orientations by measuring stimulation-induced DBS magnetic fields, which overcome the fundamental drawbacks of current postoperative imaging techniques without exposing patients to radiation at all. The feasibility of DBS electrode localization based on scalp EEG has already been demonstrated, but localization errors of more than 10 mm have been reported [17]. The potential of MEG recordings to detect electrode position and orientation has been demonstrated in our previous research [18,19,20,21,22]. In this study, we present an improved and patient-oriented method for the radiation-free detection of electrode positions and orientations using a series of measurements with a conventional SQUID-based MEG. This method has been given the name MaDoPO (Magnetic Detection of Position and Orientation). For this study, a head–torso phantom with realistic dimensions was constructed, and the DBS electrode was placed at a realistic position in the phantom’s head. Postoperative CT was performed to determine the position and orientation of the electrode using state-of-the-art neuroimaging approaches and to compare them with those obtained from MEG recordings. A series of measurements were made with the MEG system under different directional and non-directional bipolar electrode configurations to find the measurement sequence that achieved clinically reliable accuracy with the smallest number of recordings. Although SQUID-MEG systems are not widely available, new technologies toward variable on-scalp or cap-shaped MEGs, which are currently the subject of intense research [23,24,25], will make magnetic detection of electrode positions and orientations feasible in daily routines. Whether such novel systems could improve detection accuracy is also being investigated in this study.

## 2. Materials and Methods

**Figure 1:**
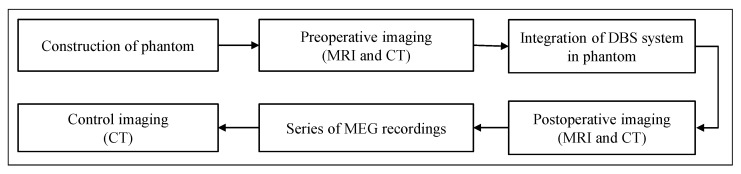
Steps performed to generate imaging and MEG measurement data as part of this study. Preoperative imaging was used to create a 3D model of the phantom. Postoperative imaging was used to determine electrode positions and orientations using a state-of-the-art approach. Localization and orientation detection results from MEG recordings were compared with those from postoperative imaging.

Figure 1 provides an overview of the sequence of data generation steps performed within this study. Once the realistic head phantom was constructed, preoperative imaging (both MRI and CT) was performed. Then, the DBS system, consisting of a directional electrode and a current-controlled implanted pulse generator (IPG), was inserted and fixed inside the phantom. A DBS neurosurgeon determined the position of the small opening on the head’s surface for insertion of the electrode and the depth of electrode placement. Postoperative imaging (both MRI and CT) was then performed. These steps were performed at the Faculty of Engineering (construction of the phantom) and at the Faculty of Medicine (imaging) at Kiel University. The series of MEG measurements with different electrode configurations were performed at the Faculty of Medicine in Düsseldorf University, where a control CT was also performed directly afterwards to check and exclude possible displacement of the electrode. Preoperative imaging was used to create a 3D model of the phantom for precise electromagnetic modeling. Postoperative imaging was used to localize the electrode and determine the electrode’s orientation (using state-of-the-art commercial Guide™ XT software) and to compare the results obtained with those of our MEG-based MaDoPO method. The individual steps from Figure 1 are described in more detail in the following subsections.

### 2.1. Phantom Design

**Figure 2:**
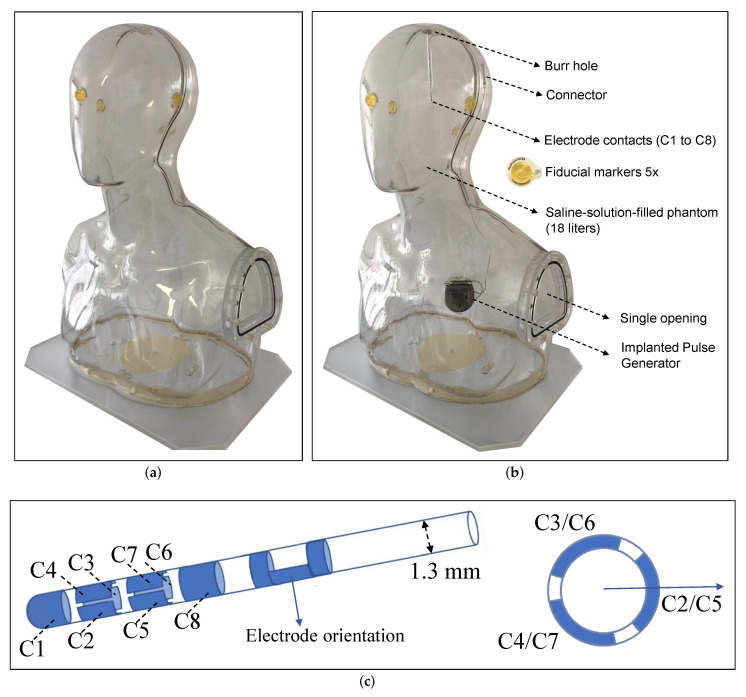
(**a**) Phantom with five fiducial markers attached to the surface of the phantom’s head before DBS intervention. (**b**) Phantom with integrated DBS system consisting of a directional electrode, extension, and Implanted Pulse Generator. (**c**) Directional electrode in side view (left) and bottom view (right), showing the eight numbered contacts (C1–C8), and how the middle two contact levels are divided into three equally spaced segments. The arrow indicates electrode orientation.

The phantom designed for this study is depicted in Figure 2a, which includes both the head and the torso to approximate a human body. The dimensions of this phantom were as follows: 55 cm height, 35 cm width, and 25 cm depth. The phantom was made of acrylic glass and was therefore neither electrically conductive nor magnetic. It was filled with approximately 18 liters of isotonic fluid (NaCl 0.9%) through the only opening on the phantom, which was used to provide electrical conductivity between electrode contacts and the implanted pulse generator (IPG). Five fiducial markers (PINPOINT-128, Beekley Medical^®^) were placed at three anatomical landmark points (nasion, right pre-auricular (RPA), and left pre-auricular(LPA)) and on the top and back of the phantom to ensure accurate co-registration between the different modalities. The phantom, as it appeared following DBS intervention, is illustrated in Figure 2b.

A DBS system from the Boston Scientific Corporation (Boston Scientific, Marlborough, MA, USA), consisting of a current-controlled IPG (Vercise™ PC) and a directional electrode (Versice Cartesia™), was chosen, as it is currently in routine clinical use. This system is based on stimulation with rectangular pulses comprising a stimulation pulse phase and a passive charge-balancing phase. The position of the burr hole, the position and implantation angle of the electrode, the position of the connector and IPG, the course of the wire, and the drilling of the excess lead wire were determined by our DBS neurosurgeons and taped in the appropriate locations. To prevent the electrode from moving inside the phantom, it was glued into a transparent, rigid plastic tube glued in the burr hole, with only the electrode contacts and the CT radiopaque marker on the electrode protruding from the top of the phantom.

The directional electrode consists of eight individually controlled platinum–iridium contacts (C1–C8), with the two middle contact levels divided into three segments, each covering 90° of the circumference (with 30° spacing between adjacent segments) as shown in Figure 2c. Any combination of these contacts can be activated to control the direction of the stimulation current. This technology, also known as Multiple Independent Current-Controlled (MICC) technology, has allowed us to precisely assign current amplitudes to the contacts regardless of impedance differences [26] and to set any desired electrode configuration inside the phantom. In this study, we investigated four different types of configurations that differed in the direction of the current flow and, therefore, in the distribution of the magnetic field. More details are discussed under ‘MEG Data Acquisition’ in Section 2.4.

### 2.2. Neuroimaging of the Phantom

**Figure 3:**
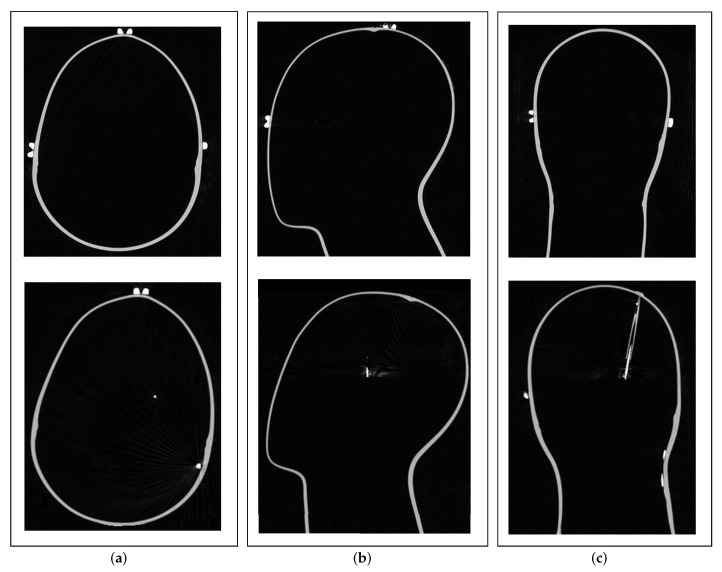
(**a**) Axial, (**b**) sagittal, and (**c**) coronal views of preoperative (upper row) and postoperative (lower row) CT imaging of the phantom. Preoperative imaging was used to create a 3D model of the phantom for an accurate electromagnetic model. Postoperative imaging was used to compare the results in electrode position and orientation detection with results from magnetic detection.

Before the DBS system was integrated into the phantom, MRI and CT examinations of the phantom were performed as normal for patients. The upper row in Figure 3 shows the preoperative CT of the phantom in axial, sagittal, and coronal view. The shape and geometry of the head and the position of the fiducial markers are clearly visible. From these images, an accurate 3D model of the phantom was created by segmenting the surface of the phantom’s head, which was used to create a precise electromagnetic model. Details of this can be found under ‘Modeling’ in Section 2.7. These images were also used by our DBS neurosurgeons to plan the DBS surgery, both the position of the burr hole on the surface of the phantom’s head and the final position of the electrode to be implanted were determined. Thus, it can be assumed that the position and implantation angle of the electrode match those of real DBS patients. The lower row in Figure 3 depicts the postoperative CT in the three dimensions. The transparent plastic tube through which the electrode was passed can be seen in Figure 3c. Postoperative imaging was performed according to Brainlab AG (Munich, Germany) scanning recommendations to ensure precise detection of DBS electrode position and orientation using commercial, state-of-the-art Guide™ XT software. After transportation and MEG measurements, a control CT of the phantom was performed to detect any displacement or rotation of the electrode during the study using the same software. No displacement or rotation was detected.

### 2.3. MEG Preparation

**Figure 4:**
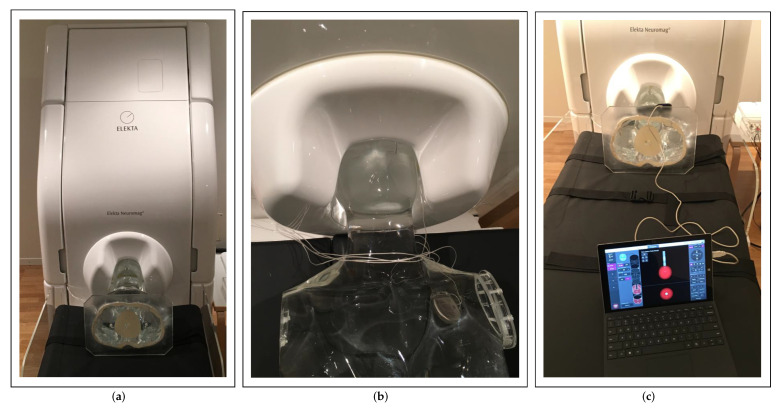
(**a**) Phantom placed on patient bed in the MEG scanner. (**b**) Phantom head within the MEG sensor array. Lower part of the electrode, IPG, and coil cables are visible. (**c**) Programming of the IPG using a programming device.

Several preparatory steps were performed before starting the actual MEG recordings. Four head position indicator (HPI) coils were placed at recommended positions on the surface of the phantom’s head, i.e., two coils behind the ears and two coils on the upper forehead. The most commonly used digitizing system (Fastrak, Polhemus Inc., Colchester, VT, USA) was then used to first localize anatomical reference points, i.e., the center holes of Nasion, RPA, and LPA fiducial markers, and subsequently the HPI coils. The digitizing system uses an alternating current electromagnetic transmitter and receiver to digitize the positions of the points in space. The device has a static position accuracy of 0.8 mm and an orientation accuracy of 0.15° [27]. Furthermore, 500 additional points were localized around the phantom to improve co-registration accuracy with imaging. The phantom was then placed in the MEG scanner and not moved until the end of the measurements as illustrated in Figure 4a,b. In such conventional MEG scanners, the positions and orientations of the MEG sensors relative to each other are fixed and known beforehand, so that only the localization of the phantom relative to the sensor array is required. Excitation of the HPI coils at different times and frequencies and detection of the distribution of the magnetic fields they generate by the MEG system allowed localization of the coils relative to the MEG sensor array. The minimum errors in the distances between the fitted coil positions and the digitized positions were as follows: HPI pair 2–3: −0.6 mm, HPI pair 2–4: −0.4 mm, and HPI pair 3–4: 0.4 mm. HPI pairs with coil 1 were not considered by the MEG system due to implausible fitting results. From these errors, it can be concluded that the accuracy in detection of the phantom position in the MEG scanner was limited to 0.6 mm, which was given by the error between the HPI pair 2–3. The sum of all inaccuracies in the system, ±0.8 mm (digitization accuracy) and ±0.6 mm (HPI fitting accuracy), resulted in a value of ±1.4 mm, which described the spatial resolution of the MEG system. This value described a crucial factor with respect to electrode localization and orientation detection, as it limits detection accuracies.

### 2.4. MEG Data Acquisition

**Figure 5:**
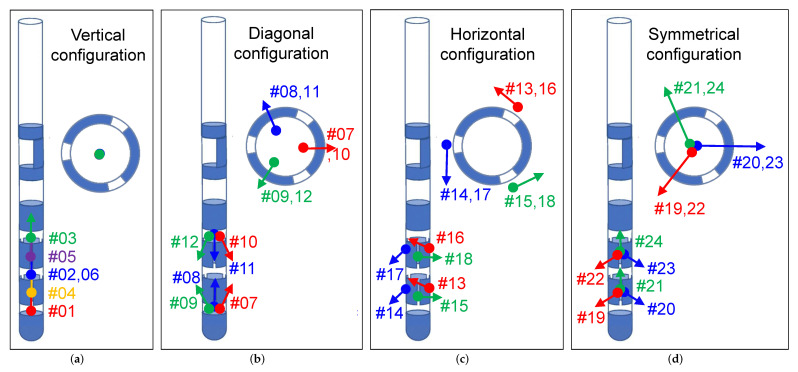
Four different electrode configuration types were analyzed: (**a**) vertical, (**b**) diagonal, (**c**) horizontal, and (**d**) symmetrical electrode configurations, which differ in the physical center of stimulation (thick colored dots) and the direction of current flow (colored arrows).

MEG data were collected using the Elekta Neuromag VectorView^®^ MEG scanner. The scanner consisted of 306 individual channels corresponding to 102 high-sensitivity Superconducting QUantum Interference Device (SQUID) magnetometers and 204 SQUID gradiometers. Only the magnetometer data were used in this study. The noise level of the magnetometer sensors was measured at approximately 3 fT$/\sqrt{\mathrm{Hz}}$ (as also indicated in the data sheet) at the beginning of the MEG recordings as part of a reference measurement. These sensors were ideally suited to measure the magnetic fields generated during electrical stimulation, which were in the range of a few picoTesla. The acquisition parameters of the MEG scanner were set as follows: The sampling rate was set to its maximum of 5 kHz. The low-pass filter was set to its highest possible value of 1660 Hz, and the cutoff frequency of the high-pass filter was set to DC (no high-pass filtering) to measure the magnetic field generated by the stimulation with the maximum allowable acquisition bandwidth, since the DBS signal included the adjusted stimulation frequency and its next harmonic frequencies. The duration of each measurement was 180 s. A total of 24 measurements were taken using bipolar electrode configurations with clinically used DBS stimulation parameters with 3 mA amplitude, 60 μs pulse width, and 130 Hz frequency (Figure 4c). These values are normally used in routine clinical practice. The performed measurements differed by the activated electrode contacts as listed in Table 1, which we divided into four vertical, diagonal, horizontal, and symmetrical configuration types. The numbers C1 to C8 in the table represent the electrode contact numbers (according to Figure 2c), where the first number represents the cathode and the second number represents the anode. The motivation for the choice of used electrode configurations is summarized in Figure 5 and can be motivated as follows: The first six measurements (#01–#06) described all possible measurements performed with non-directional electrode configurations, in which all three segmented contacts on the two middle levels of the electrode were activated with the same amount of current to allow conventional ring stimulation (a 33/33/33 ratio of current is guaranteed by MICC technology). These measurements were used to investigate electrode localization. In the ‘vertical’ electrode configuration, the physical center of stimulation (represented by thick colored dots) lay exactly between activated contacts, e.g., between contacts C1 and C234 in the first measurement, with the stimulation current flowing from the cathode to the anode, thus exhibiting a vertical current flow direction along the longitudinal axis of the electrode (indicated by the colored arrows). The remaining measurements, performed with different directional electrode configurations (#07–#24), were used to investigate electrode orientation detection. In the ‘diagonal’ configuration, the physical center of stimulation was located deeper within the electrode, e.g., between contacts C1 and C2 in measurement #07, and the current flow from the cathode to the anode there exhibited a diagonal current flow direction. In the ‘horizontal’ configuration, the physical center of stimulation lay exactly between adjacent segmented contacts, e.g., between contacts C2 and C3 in measurement #13, and the current flowing from the cathode to the anode there exhibited a horizontal current flow direction (perpendicular to the longitudinal axis of the electrode). In the ‘symmetrical’ configuration, two adjacent segments were stimulated in a 50/50 ratio against the third segment on the same level of the electrode so that the current flow propagates symmetrically around the electrode from both cathode contacts to the anode contact (perpendicular to the longitudinal axis). The current, the direction of which was defined by the corresponding configuration, generated a defined magnetic field. The magnetic field distribution was measured around the phantom by the MEG sensor array and used with precise electromagnetic simulations and detection algorithms to derive electrode positions and orientations. Since the measurements in this study were performed under ideal conditions with a phantom, the measured MEG signals were not contaminated by subject-related biological artifacts, such as cardiac activity, skeletal muscles, eyes, head, and body movements. Ambient noise consisted solely of power line interference, which affected only insignificant frequencies (50 Hz and harmonics).

### 2.5. The Pipeline of the MaDoPO Method

**Figure 6:**
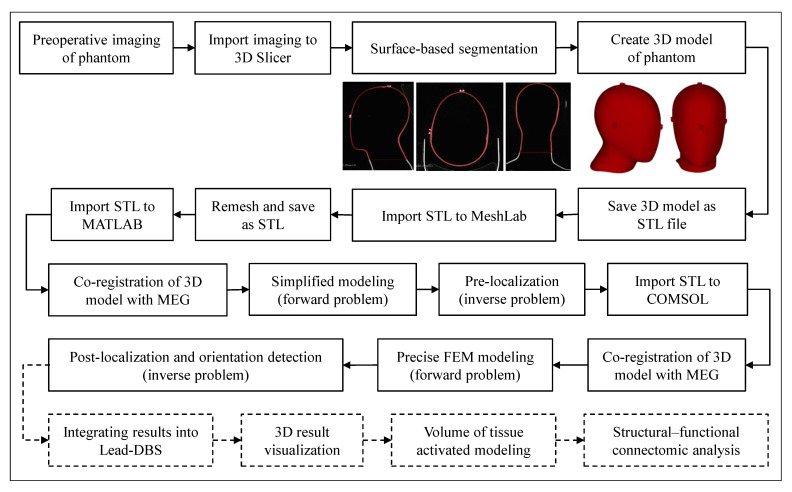
Steps in the modeling process: Preoperative CT is imported into 3D Slicer software, where surface-based segmentation is performed to create the 3D model of the phantom. The model is decimated by remeshing it using MeshLab software. The model is then imported into the appropriate software for modeling.

Figure 6 provides an overview of the procedure for MEG-based localization of the electrode and detection of the electrode orientation in the phantom, which could also be performed in real-patient analysis. First, the preoperative imaging (CT in this case) was imported into 3D Slicer software, which is a free open-source medical imaging software. The surface-based segmentation was performed using this software, first by applying the ‘threshold segmentation’ function to obtain the preliminary results of the automatic segmentation. Then, manual correction was performed slice by slice, and the result was finally smoothed. After that, a 3D model of the phantom was created and was saved as a STL (STereoLithography) file, a triangulated representation of a 3D model. The file contained the following mesh information: vertices 2,537,752, faces 5,076,736, and storage space approximately 250 MB. Since the mesh resolution was too high for this application, which would have resulted in far too much computation time for modeling, the model was imported into MeshLab, an open source system for processing and editing 3D meshes, and decimated by remeshing. This resulted in the following mesh information: 20,353 vertices, 40,702 faces, and approximately 2 MB of storage space. This file was first imported into MATLAB software to perform a pre-localization of the electrode. In this process, the created 3D model, generated based on CT data, and thus defined in CT coordinates, had to be transformed into the coordinate system of the MEG system. As mentioned in Section 2.3, the coordinate system in which the MEG sensors were expressed was defined based on three anatomical landmarks identified on the phantom’s head (Nasion, LPA, and RPA). FieldTrip Toolbox was used to co-register the anatomical image with the MEG sensor array, which was performed using the ‘ft_volumerealign’ function [28]. However, the positions of the anatomical landmarks in the CT data had to be given as inputs, which could be accurately localized by the center hole of the attached fiducial markers. The aim of pre-localization was to estimate the position of the electrode in a short time and to narrow down the volume for post-localization by defining the region of interest (ROI). For this purpose, it was sufficient to create a simplified model (i.e, a model of an electric current dipole). Then, the 3D model was imported into the COMSOL software and aligned to the MEG coordinate system based on the translation and rotation results of the co-registration performed previously. An accurate finite element electromagnetic model was created and used together with processed MEG recordings for electrode post-localization and orientation detection. The main steps are described in detail in the following sections. As illustrated with dashed lines in Figure 6, future perspectives of our method include the integration of the localization and orientation detection results into the open-source Lead-DBS software [29,30] to use its implemented features, such as 3D visualization of the electrode within patient-specific anatomy, modeling of the volume of tissue activated based on the applied stimulation parameters, and structural–functional connectivity analysis if needed.

### 2.6. Signal Processing of Measured Data

**Figure 7:**
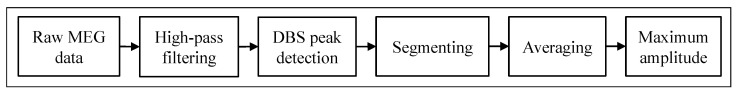
Signal processing pipeline: Each measured MEG signal was high-pass filtered with a 60 Hz cutoff frequency, segmented into short DBS periods (according to the locations of DBS peaks detected by a peak detection algorithm), and averaged to a single time period. The maximum amplitude value was then taken.

The processing steps used on the measured MEG data are provided as an analysis pipeline in Figure 7. For each measurement, we obtained 102 time signals, one from each MEG sensor. The measured data were imported into MATLAB (version R2018a) and pre-processed using the FieldTrip toolbox [28]. Each time signal, which was 180 s long, was high-pass filtered using a sixth-order Butterworth filter with a cutoff frequency of 60 Hz without losing signal components, since the stimulation signal contained much higher frequencies (stimulation frequency of 130 Hz and its harmonics). Then, a peak detection algorithm was used to detect the location of successive DBS peaks in the time signal, and, in the next step, each signal was divided into individual DBS time segments. These segments were then averaged, which significantly improved the signal-to-noise ratio (SNR) by $\sqrt{N}$ with $N=180\;s\cdot 130\;s^{-1}$ averages. The maximum value from this averaged time segment (at a specific time point with the largest SNR) was then taken for each MEG sensor, resulting in a total of 102 values, with $B_{\mathrm{meas}}$ representing the measured magnetic field distribution of the corresponding MEG recording. Since the overall background noise over all frequencies and especially over the frequencies of interest (stimulation frequency and its harmonics) was so low that the magnetic field generated by the stimulation could be easily measured, no further signal processing steps such as artifact rejection methods (e.g., signal space separation) needed to be used.

### 2.7. Modeling

A simplified model was used for electrode pre-localization and precise finite element electromagnetic model was used for electrode post-localization and orientation detection.

#### 2.7.1. Simplified Model

The simplified model includes the model of an electric current dipole, which can be calculated using the following Biot–Savart law [31]:

(1) $$B_{\mathrm{pre},\mathrm{model}}\left(pd,ad\right)=\frac{\mu_{0}}{4\pi }\frac{\left(\vec{R}-{\vec{L}}_{pd}\right)\times \hat{s}\cdot \vec{Q}}{|\vec{R}-{\vec{L}}_{pd}{|}^{3}},$$

where pd is the dipole position, ad is the dipole orientation, $\vec{Q}$ is the dipole moment with $\vec{Q}=I\cdot {\hat{s}}_{ad}$, *I* is the stimulation current, ${\hat{s}}_{ad}$ is the direction of current flow, $\vec{L}$ is the dipole location, $\vec{R}$ is the sensor location, and $\hat{s}$ is the unit orientation of the sensor. The entire volume of the phantom’s head was divided into a regular coarse grid with a size of 90 × 90 × 90 mm and a resolution of 10 mm, resulting in a total number of $N_{pd}=1000$ dipole positions. For each dipole position, a dipole orientation oriented in the *z*-direction was tilted in the *x*- and *y*-directions in 5° steps between 0° and 30° and rotated in 45° steps on a circle between 0° and 360°, resulting in a total number of approximately $N_{ad}=70$ dipole orientations. For each different position–orientation pair, the magnetic field was calculated using the above equation. In previous papers [18,19], we have demonstrated that this dipole model, which applies to an infinite homogeneous space and neglects the modeling of the volume conduction, describes a good approximation for the bipolar electrode configuration depicted in Figure 5 within realistic distances between electrode and MEG sensors. Although this model cannot be used for precise millimeter-accurate localization (since it is only a simplification of the real world), it is sufficient for pre-localization to estimate in which area the electrode is approximately located without much computational time, so that the actual millimeter-accurate search can be performed with the precise electromagnetic finite element model, which requires much more effort and computational time. Post-localization is performed within the ROI with dimensions 29 × 29 × 29 mm with 1 mm resolution, whose position is defined by the result of pre-localization. The associated simulations of the dipole model were performed using MATLAB.

#### 2.7.2. The Finite Element Electromagnetic Model

**Figure 8:**
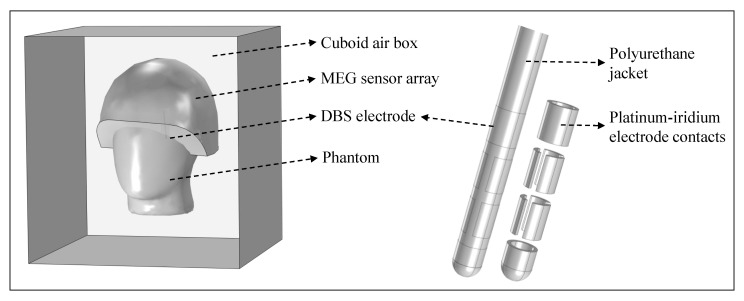
Finite element electromagnetic model. It includes the phantom filled with saline solution, the directional electrode placed inside the phantom, and the helmet-shaped MEG sensor surface. These structures are surrounded by a cuboid air box.

**Figure 9:**
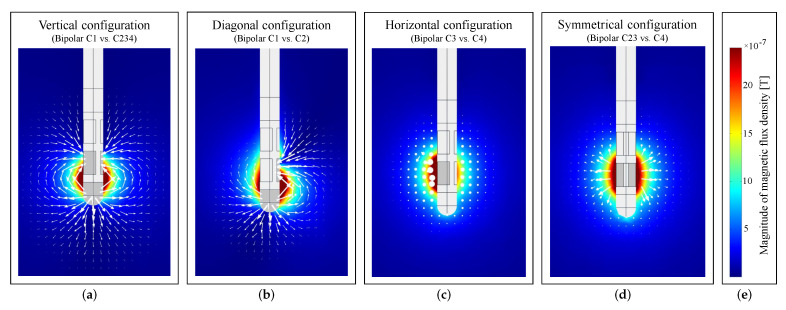
Electromagnetic simulation results of a (**a**) vertical, (**b**) diagonal, (**c**) horizontal, and (**d**) symmetrical electrode configuration, in which the magnetic field is shown in color and the direction of current flow is shown with white arrows. (**e**) Color legend applies to all images.

The electromagnetic finite element simulations were performed based on a model created in COMSOL Multiphysics^®^, which is shown in Figure 8. For each structure in the model, the corresponding material was taken directly from the built-in material library, and values were assigned for uniform electrical conductivity σ, relative permeability $\mu_{r}$, and relative permittivity $\epsilon_{r}$. The phantom and the outer jacket of the electrode were modeled with acrylic plastic (σ≈0S/m, $\mu_{r}=1$, $\epsilon_{r}=4.2$), the eight independent electrode contacts with platinum–iridium ($\sigma =10^{6}\;S/m$, $\mu_{r}=1$, $\epsilon_{r}=1$), and the content of the phantom with saline solution (σ=1.6S/m, $\mu_{r}=1$, $\epsilon_{r}=80$). The phantom, whose 3D model was imported into COMSOL and moved to the MEG-based position according to the MEG-CT co-registration result, and the modeled directional electrode were surrounded by a cuboid box with a length of 500 mm filled with air (σ≈0S/m, $\mu_{r}=1$, $\epsilon_{r}=1$). The MEG helmet was modeled as a surface and all measurement points were placed on that surface. A tetrahedral mesh with ‘Extra Fine’ resolution was used. To model the bipolar electrode configurations from Table 1, the boundary condition ‘Terminal’ of ‘Current Type’ was used for the cathode and the boundary condition ‘Ground’ was used for the anode. The simulations were performed using the ‘Magnetic and Electric Fields (mef)’ interface of the ‘AC/DC module’ of COMSOL. Figure 9 illustrates the electromagnetic simulation results of the vertical, diagonal, horizontal and symmetrical electrode configuration considered in this work. The corresponding magnetic field distribution is color coded, and the direction of current flow is marked by white arrows. The magnetic flux densities in the *x*-, *y*-, and *z*-directions ($B_{x}$, $B_{y}$, and $B_{z}$) on the surface of the MEG helmet were calculated. In this experiment, the MEG sensors measured the normal field component of the magnetic field perpendicular to the helmet surface. To determine the magnetic field $B_{\mathrm{post},\mathrm{model}}\left(pe,ae,ce\right)$ perpendicular to the helmet surface in the simulation, the dot product $B_{\mathrm{post},\mathrm{model}}=\vec{B}\;\cdot \;\vec{s}$ was calculated, where $\vec{s}$ is the unit vector perpendicular to the helmet and $B=B_{x}+B_{y}+B_{z}$. Thus, the modeled magnetic field depended on the position of the electrode pe, the orientation of the electrode ae, and the applied electrode configuration ce, with the electrode taking only the positions in 1 mm steps in the predefined ROI with the dimensions 29 × 29 × 29 mm (number of electrode positions in the model $N_{pe}$ = 27,000). For electrode post-localization of each electrode configuration considered in this study, the position of the electrode was moved within the predefined ROI in the model in 1 mm steps, and the expected magnetic field values at the MEG sensor positions were calculated. In determining electrode orientation, it was assumed that electrode localization had already been performed and that the position of the electrode was precisely known. In the model, therefore, the electrode was placed at the appropriate location and rotated around its own longitudinal axis in 1° steps, allowing a total of $N_{rot}=360$ orientations. The expected magnetic field, $B_{\mathrm{model}}\left(rot,ce\right)$, depended only on the rotation of the electrode, rot, and on the electrode configuration, ce.

### 2.8. The Method Used for Electrode Localization

For electrode localization, the measured and processed values, according to the signal processing pipeline in Figure 7, were compared with the modeled values. As stated, all modeled data described the magnetic field values that would be measured by the MEG sensor array if the electrode occupied the defined position in the phantom. To ensure comparability between measured and modeled data, the data were normalized to the respective absolute maximum values beforehand and the Goodness of Fit (GoF) was calculated between each measured and modeled data point using the normalized root mean square error (NRMSE) as a cost function using Equations (2) and (3) for pre-localization and post-localization, respectively, as follows:

(2) $${\mathrm{GoF}}_{\mathrm{meas},x}=1-\frac{\left|\right|B_{\mathrm{pre},\mathrm{model}}\left(pd,ad\right)-B_{\mathrm{meas},x}\left|\right|}{\left|\right|B_{\mathrm{pre},\mathrm{model}}\left(pd,ad\right)-\mathrm{mean}\left(B_{\mathrm{pre},\mathrm{model}}\left(pd,ad\right)\right)\left|\right|}$$

(3) $${\mathrm{GoF}}_{\mathrm{meas},x}=1-\frac{\left|\right|B_{\mathrm{post},\mathrm{model}}\left(pe,ae,ce\right)-B_{\mathrm{meas},x}\left|\right|}{\left|\right|B_{\mathrm{post},\mathrm{model}}\left(pe,ae,ce\right)-\mathrm{mean}\left(B_{\mathrm{post},\mathrm{model}}\left(pe,ae,ce\right)\right)\left|\right|},$$

where a GoF value of 1 indicates a perfect fit, and ||.|| is the 2-norm of a vector. The estimated electrode position was then given with the minimum NRMSE value or the maximum GoF value for the particular measurement *x*. The electrode localization error was calculated using the Euclidean distance between the estimated electrode position in the model and the real, known position of the electrode in the phantom, which was determined using a state-of-the-art neuroimaging approach. To improve localization accuracy, all measurements with non-directional electrode configuration (measurements with vertical electrode configuration from Table 1) were fitted and evaluated together, since the solution of the inverse problem could be constrained by providing a priori known information about the electrode geometry (i.e., the distance between different contacts of the electrode):

(4) $$\begin{array}{c} \mathrm{GoF}=1/6\cdot \sum_{x=1}^{6}{\mathrm{GoF}}_{\mathrm{meas},x}\to \min. \end{array}$$

The electrode localization results from the magnetic detection were compared with the co-registered results obtained from postoperative CT imaging using GuideXT software. It should be noted that localization errors may occur due to the discretization of the localization area. The Euclidean distance between both results is defined as the localization error. If the sensor is not directly on a defined grid point, the localization error is at least the distance between the nearest grid point and the location of the sensor. To solve this problem, a higher resolution is required. However, this would lead to a drastic increase in computational complexity during post-localization. A lower initial grid resolution during pre-localization was used to keep the computational complexity low (a few minutes). Precise electromagnetic modeling with a resolution of 1 mm within the ROI for post-localization required a pure computational time of approximately two weeks, i.e., a resolution of 0.5 mm would increase the computational time by a factor of eight (equivalent to 16 weeks). Simulations were performed on a 64-bit computer configured with anIntel core i7, 3.6 GHz processor with 32 GB RAM.

### 2.9. The Method Used for Electrode Orientation Detection

Similar to electrode localization, for electrode orientation detection, the measured and processed values are compared with the modeled values. Each modeled data point describes the magnetic field values that would be measured by the MEG sensor array if the electrode occupied its defined orientation within the phantom. To ensure comparability between the measured and modeled data, the data were normalized to their respective absolute maximum values beforehand, and the GoF was calculated between each measured and modeled data point using the NRMSE as a cost function, as follows:

(5) $$\begin{array}{c} {\mathrm{GoF}}_{\mathrm{meas},x}=1-\frac{\left|\right|B_{\mathrm{model}}\left(rot,ce\right)-B_{\mathrm{meas},x}\left|\right|}{\left|\right|B_{\mathrm{model}}\left(rot,ce\right)-\mathrm{mean}\left(B_{\mathrm{model}}\left(rot,ce\right)\right)\left|\right|}. \end{array}$$

The estimated result for the electrode orientation was then given by the minimum NRMSE value or by the maximum GoF value for the particular measurement *x*. The error in determining the electrode orientation was calculated using the absolute angle between the electrode orientation in the found model and the real known orientation of the electrode in the phantom obtained by the state-of-the-art neuroimaging approach. To improve electrode orientation detection accuracy, different measurements with directional electrode configuration were fitted and evaluated together, since the solution of the fitting approach could be constrained by providing a priori known information about the electrode geometry and corresponding electrode configurations (i.e., the angle between each segmented contact of the electrode):

Considering measurements within same electrode configuration:

(6) $$\begin{array}{c} {\mathrm{GoF}}_{\mathrm{config}}=1/N_{config}\cdot \sum_{x=1}^{N_{config}}{\mathrm{GoF}}_{\mathrm{meas},x}\to \min. \end{array}$$

Considering measurements within multiple electrode configurations:

(7) $$\begin{array}{c} {\mathrm{GoF}}_{\mathrm{multip}}=1/N_{multip}\cdot \sum_{x=1}^{N_{multip}}{\mathrm{GoF}}_{\mathrm{config},x}\to \min. \end{array}$$

Considering all performed measurements:

(8) $$\begin{array}{c} {\mathrm{GoF}}_{\mathrm{total}}=1/N_{total}\cdot \sum_{x=1}^{N_{total}}{\mathrm{GoF}}_{\mathrm{multip},x}\to \min. \end{array}$$

The results of the electrode orientation detection with magnetic measurements were compared with the results of postoperative CT imaging using GuideXT software. For this purpose, in the software, an anterior to posterior commisure (AC-PC) line was first defined, with the AC point determined by localizing the center hole of the nasion fiducial marker and the PC point determined by the center hole of the back fiducial marker. Relative to the mid-commisural (MC) point, the orientation of the electrode was then determined, with both 99° and −89° being the results of orientation detection. The difference in absolute angle between both real and calculated orientation is defined as the orientation detection error.

## 3. Electrode Localization Results

The following Cartesian coordinates of the electrode were determined within the MEG coordinate system by the state-of-the-art approach: Electrode tip [−13.8; 14; 4] mm, mid points of C1 [−14; 14.2; 4.6] mm; C234 [−14.5; 14.9; 6.5] mm, C567 [−14.9; 15.5; 8.3] mm, and C8 [−15.4; 16.1; 10.2] mm.

### 3.1. Electrode Pre- and Post-Localization

**Figure 10:**
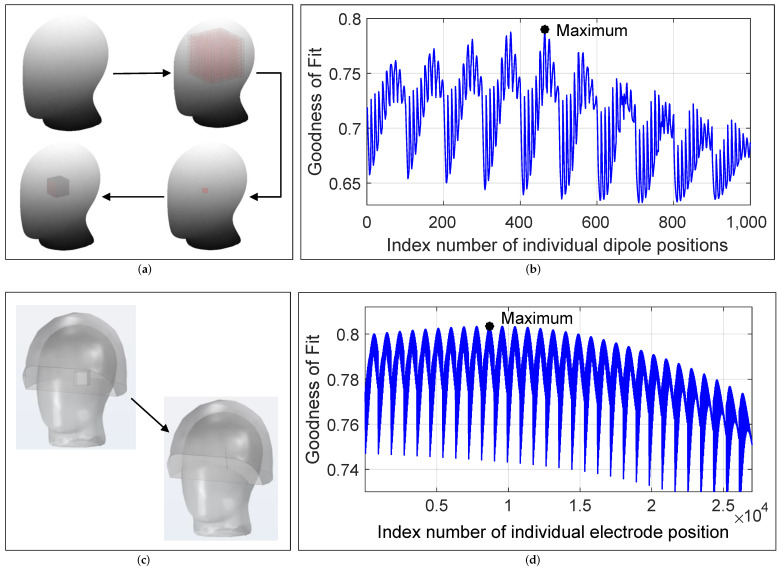
(**a**,**c**): Schematic representation of the procedure for pre- and post-localization of the electrode. (**b**,**d**): GoF values between modeled and measured data, with 1000 positions for the dipole in the simplified dipole model and 27,000 positions for the electrode in the electromagnetic model.

For electrode pre-localization, a volume with dimensions 90 × 90 × 90 mm was first defined in the phantom’s head with a coarse resolution of 10 mm, as shown in Figure 10a. Then, the processed measured data from the first measurement in Table 1 were compared with the modeled data from the dipole model with the GoF value for each (numbered) grid point calculated according to Equation (2). The grid point numbered 464, with coordinates [*x*; *y*; *z*] = [−10; 10; 10] mm yielded the maximum GoF value of approximately 0.78 among all 1000 dipole positions as shown in Figure 10b. Since this was a rough estimate, a cuboid of 30 mm length was placed around the localized grid point that defined the ROI for the following precise post-localization of the electrode. For this purpose, the consideration of a single measurement was sufficient. Then, the modeled electrode was moved within the ROI (see Figure 10c) in the precise electromagnetic model with 1 mm steps, and the GoF value was calculated based on the joint consideration of all non-directional measurements using Equation (4). The electrode position (or the position of the electrode tip), numbered 8689 with the coordinates [−15; 15; 4] mm, obtained the maximum GoF value of slightly above 0.8, which described the result of electrode localization (see Figure 10d). A comparison with the real electrode position revealed the following localization errors: Electrode tip 1.65 mm, C1 1.8 mm, C234 2.01 mm, C567 2.26 mm, and C8 2.39 mm, providing an averaged error of 2.02 mm. It can be argued that the localization error of approximately 2 mm was due to several inaccuracies in the system, with the spatial resolution of the MEG scanner being the largest contributor. Assuming a MEG spatial resolution error of 1.4 mm, as described in detail in Section 2.3, it can be concluded that the remaining error of approximately 0.6 mm arose appropriately elsewhere during the localization process, for example, due to inaccuracies in the measurements (noise) and simulations (material parameters).

### 3.2. Localization Accuracy and Number of Sensors

**Figure 11:**
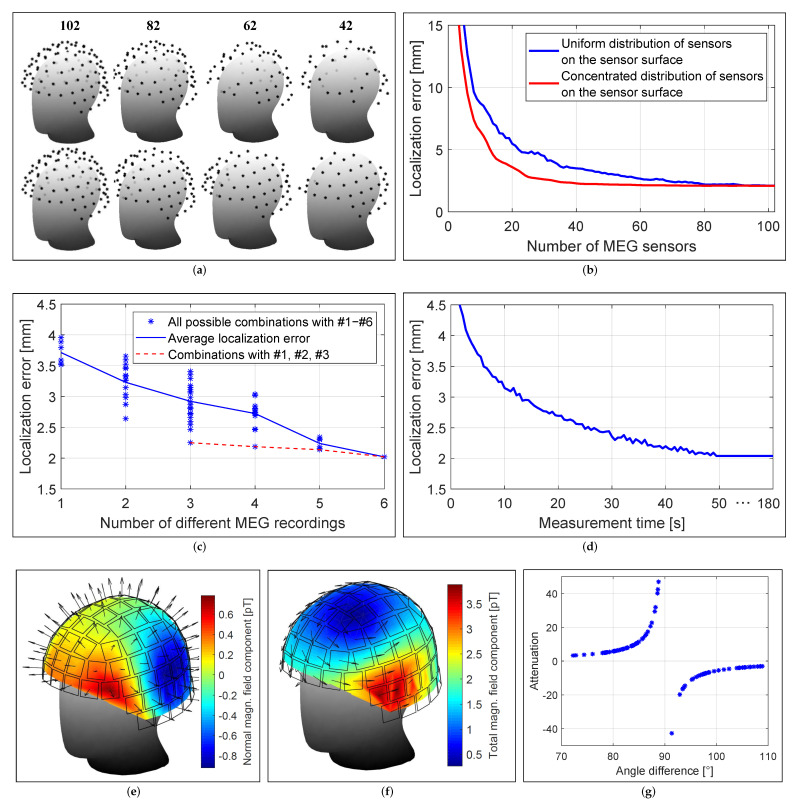
(**a**) Distribution of different numbers of MEG sensors on the sensor surface. Localization error as a function of (**b**) the number of MEG sensors and their distributions, (**c**) the number of MEG recordings with different non-directional stimulation modes, and (**d**) the recording time for each measurement. (**e**) Measured magnetic field distributions with a non-directional stimulation configuration. Arrows indicate the orientation of the MEG sensors. (**f**) Expected magnetic field distribution for the magnitude of the magnetic field. Arrows indicate the direction of the magnetic field. (**g**) Attenuation of measured magnetic fields due to the angle between the orientation of the MEG sensor and that of the magnetic field.

The localization accuracy depended on the number of MEG sensors and their local distribution on the sensor surface as illustrated in Figure 11b. The conventional MEG helmet system provided high spatial resolution of the magnetic field with 102 magnetometers sampling the field distribution. If the number of sensors on the sensor surface was reduced uniformly (uniform sensor distribution), 80 sensors were also sufficient to achieve the same localization result as with 102 sensors, but the localization error increased when less than 80 sensors were considered. However, if the sensors were reduced unevenly depending on the distance of the sensor to the electrode (the sensor with the largest distance was reduced first), even 40 sensors were sufficient to achieve the same localization result. This result indicates that a concentrated distribution of sensors near the electrode is preferable to a uniform sensor distribution around the head.

### 3.3. Localization Accuracy and Number of Recordings

The localization accuracy depended on the number of measurements considered in the localization process (Figure 11c). A single measurement with a bipolar non-directional electrode configuration resulted in a maximum error of approximately 3.5 to 4 mm, while considering all possible measurements with bipolar non-directional stimulation (six measurements in total) caused a mean error of 2.02 mm. Three specific measurements with a bipolar non-directional configuration at each of the three electrode levels (C1 vs. C234, C234 vs. C567, C567 vs. C8) resulted in an error of 2.25 mm, so that the remaining three measurements had their accuracies improved by only 0.23 mm.

### 3.4. Localization Accuracy and Measurement Time

The localization accuracy depended on the measurement duration and SNR (Figure 11d). Shortening the measurement duration from 180 s (SNR gain of 21.8 dB after 23,400 averages) to 50 s (SNR gain of 19 dB after 6500 averages) resulted in no loss of localization accuracy. Shortening the measurement duration to approximately 13 s (SNR gain of 16.1 dB after 1700 averages) caused only a 1 mm degradation in accuracy. Further investigation revealed that a SNR of 20 dB (i.e., where the signal is two orders of magnitude larger than the noise) resulted in no loss of localization accuracy, whereas a SNR of 10 dB (i.e., where the signal is only one order of magnitude larger than the noise) resulted in an additional error of 1 mm.

### 3.5. Localization Accuracy and Sensor Orientation

The localization accuracy depended on the orientation of the MEG sensors. Bipolar non-directional electrode configurations produced a magnetic field that had mainly a tangential field component at the sensor surface, which can be seen as arrows in Figure 11f. However, each MEG sensor measured the normal field component, marked as arrows in Figure 11e, resulting in the measurement of an attenuated magnetic field, with attenuation ranging from a factor of 3 (4.7 dB) to 50 (17 dB), depending on the angle between the sensor orientation and the direction of the magnetic field between 70° and 110° as shown in Figure 11g. On average, a SNR gain of approximately 8.6 dB would be achieved, resulting in a significant reduction in measurement and averaging time of less than 10 s.

## 4. Electrode Orientation Detection Results

As stated in Section 2.9, the orientation of the electrode was determined to both 99° and −89° relative to the AC-PC line. With a single magnetic measurement with any directional electrode configuration, it was possible to determine that 99° corresponds to the actual orientation of the electrode.

### 4.1. Diagonal Electrode Configuration

**Figure 12:**
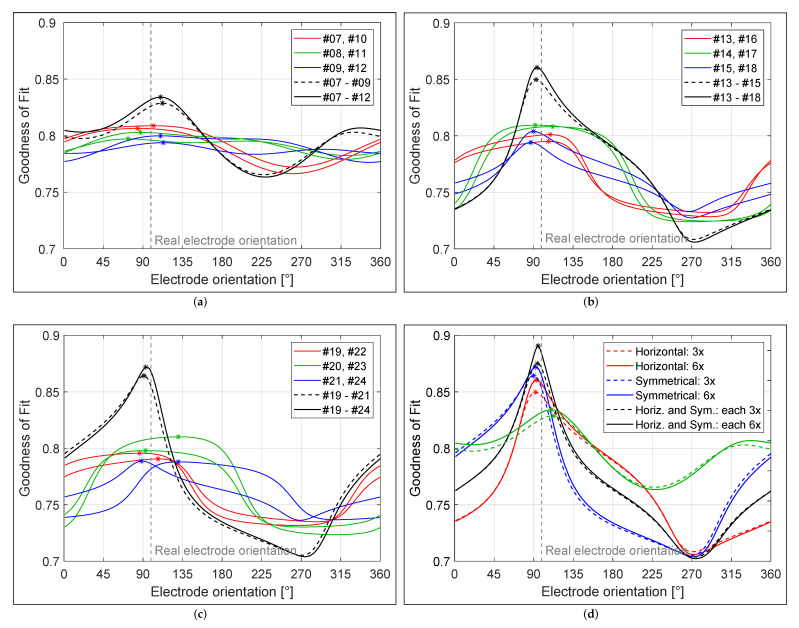
Results of electrode orientation detection for measurements with (**a**) diagonal, (**b**) horizontal, and (**c**) symmetrical electrode configurations. (**d**) Results for measurements with both horizontal and symmetrical electrode configurations.

The electrode orientation detection results of measurements with diagonal electrode configurations (i.e., measurements #7–#12) are depicted in Figure 12a. When considering individual measurements, an average accuracy of 16° was achieved with a maximum GoF value of approximately 0.8 (see colored curves). The GoF curves exhibited relatively flat behavior near the maximum, additionally allowing neighboring orientations to be considered as solutions in case of slight measurement or modeling errors. When three contiguous measurements were considered, the maximum GoF value increased to approximately 0.827, and an accuracy of 12° (determined 111° instead of 99°) was achieved. Consideration of three additional measurements resulted in a slight increase of the GoF value to approximately 0.83, and an accuracy of 10° to a slight improvement of orientation detection was achieved.

### 4.2. Horizontal Electrode Configuration

The electrode orientation detection results of measurements with horizontal electrode configurations (i.e., measurements #13–#18) are shown in Figure 12b. When considering individual measurements, an average accuracy of 11° was achieved with maximum GoF values of approximately 0.8. When three contiguous measurements were considered, an accuracy of 6° (determined to be 93° instead of 99°) was achieved, and the maximum GoF value increased to approximately 0.85. Consideration of three additional measurements resulted in only a slight improvement (maximum GoF value of 0.86 and 5° accuracy).

### 4.3. Symmetrical Electrode Configuration

The electrode orientation detection results of measurements with symmetrical electrode configurations (i.e., measurements #19–#24) are provided in Figure 12c. When considering individual measurements, an average accuracy of 15° was achieved with GoF values of approximately 0.8, with two potential outliers visible at approximately 130°. Disregarding the two outliers, an average accuracy similar to that of the horizontal configuration was observed (as expected). Since GoF curves show flat behavior around the maximum, adjacent orientations may be considered as solutions (due to measurement or modeling errors), resulting in outliers (as in this case). With three contiguous measurements, an accuracy of 8° (determined to be 91° instead of 99°) was achieved and the maximum GoF value increased to approximately 0.86. Consideration of three additional measurements resulted in only a slight improvement (maximum GoF value of 0.87 and 6° accuracy).

### 4.4. Horizontal and Symmetrical Electrode Configuration

The electrode orientation detection results considering three and six contiguous measurements for each of the three investigated electrode configurations are summarized in Figure 12d (colored solid and dashed lines). In addition, that figure presents the result of the combined consideration of the symmetrical and horizontal electrode configurations, since the combinations of both configuration types obtained the best results among all possible combinations with the diagonal configuration. The range of GoF values for the diagonal configuration was relatively flat (between 0.77 and 0.83), because the rotation of the electrode in the model had little effect on the change of the magnetic field distribution compared to other configuration types. It was found that the maximum GoF value increased to approximately 0.88–0.89 when the horizontal and symmetrical configurations were considered, depending on the number of measurements considered. An accuracy of 4° and 3° in orientation detection was achieved when three and all six measurements from each configuration were selected, respectively. This indicates that, with a total of six measurements (three each from two different configurations), an accuracy of 4° was achieved and another six measurements (three each from both configuration types) resulted in only a slight improvement of 1° in accuracy. Consideration of additional measurements with the diagonal electrode configuration did not provide any additional benefit.

**Figure 13:**
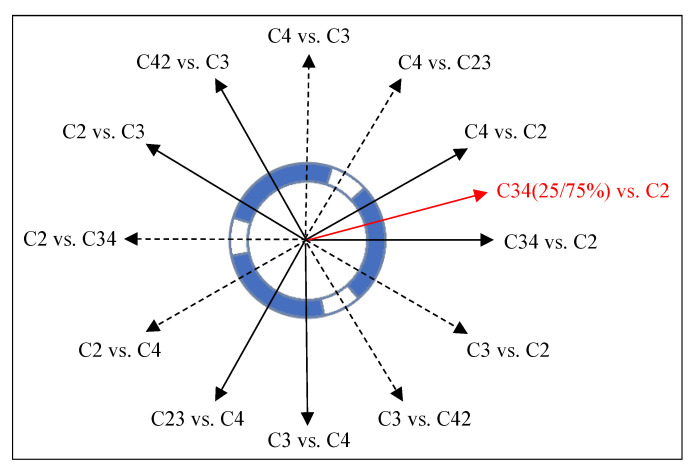
Bottom view of the electrode with arrows indicating the direction of current flow which is defined by the corresponding electrode configuration. Black solid arrows indicate the directions set by the electrode configurations investigated in this study. Dashed arrows indicate the direction of the currents by the corresponding change of anode and cathode contacts. The red arrow marks an example of a possible electrode configuration through MICC technology, which can be used to achieve finer resolution.

It was found that the horizontal and symmetrical electrode configurations provided similar results and that the combination of both types provided the best result among all possible combinations. This can be explained by the fact that both types complement each other in the direction of the current flow as illustrated in Figure 13, so that a finer sampling of the magnetic field distribution could be observed. With three measurements from each of the two configuration types, the magnetic field distribution could be measured with a resolution of 30° around the electrode. Multiple Independent Current Control (MICC) technology was developed for fine control of the stimulation position to provide very precise electrical signals through controllable current delivery and to tailor therapy to the individual patient. Thus, this technology can ensure current delivery through the electrode in any direction with the desired resolution [26]. For example, the red arrow in Figure 13 marks the current direction for a bipolar electrode configuration in which contacts C3 and C4 were activated with a 25–75% distribution of the amount of current in the direction of the third segment, C2. This would provide a 15° resolution along the electrode and presumably improve accuracy in detecting electrode orientation.

### 4.5. Orientation Detection Accuracy and Number of Sensors

**Figure 14:**
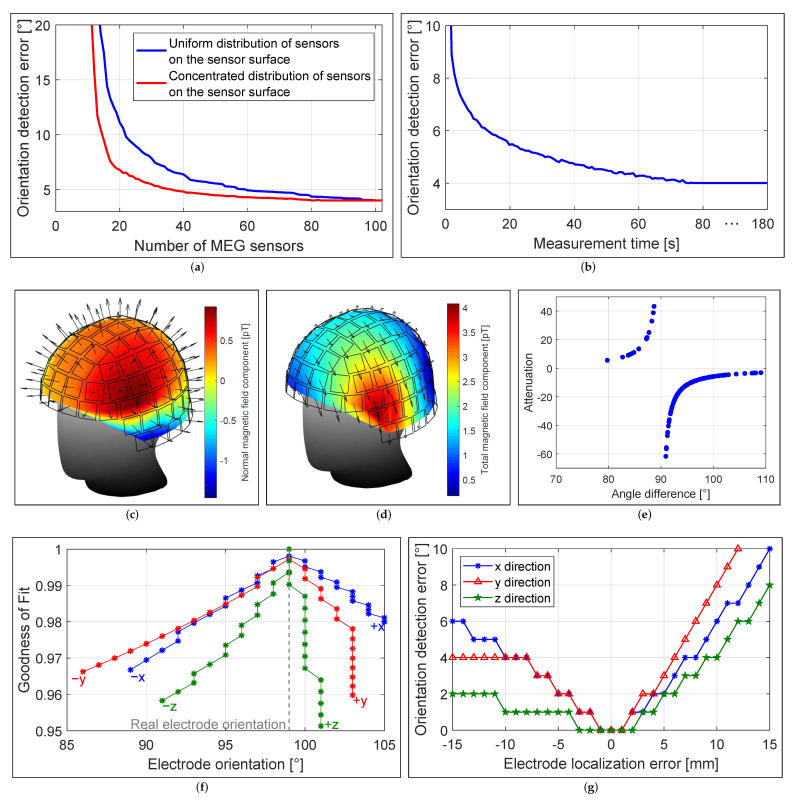
Orientation detection error as a function of (**a**) the number of MEG sensors and their distributions distributions and (**b**) the recording time of each measurement. (**c**) Measured magnetic field distribution with horizontal electrode configuration. Arrows indicate the orientations of the MEG sensors. (**d**) Expected magnetic field distribution for the magnitude of the magnetic field. Arrows indicate the direction of the magnetic field. (**e**) Attenuation of the measured magnetic fields due to the angle between the orientation of the MEG sensor and that of the magnetic field. (**f**) GoF value and (**g**) detection error as a function of electrode localization error.

The accuracy of the electrode orientation detection depended on the number of MEG sensors and their local distribution on the sensor surface as shown in Figure 14a. For this purpose, the same sensor distribution as in Figure 11a was used. When the number of sensors on the sensor surface was uniformly reduced (uniform sensor distribution), 95 sensors were also sufficient to achieve the same detection result as with 102 sensors, but the detection error increased continuously thereafter when fewer than 95 sensors were considered. However, when the sensors were reduced unevenly depending on the distance of the sensor from the electrode (the sensor with the largest distance was reduced first), even approximately 80 sensors were sufficient to achieve the same detection result. Further reduction of the number of sensors by 50 sensors led to a slight decrease in accuracy. Accuracy decreased drastically with less than 30 sensors. Similar to electrode localization, this result indicates that a concentrated distribution of sensors near the electrode is preferable to a uniform distribution of sensors around the head.

### 4.6. Orientation Detection Accuracy and Measurement Time

The accuracy of the electrode orientation detection depended on the measurement duration and SNR (Figure 14b). Shortening the measurement duration from 180 s (SNR gain of 21.8 dB after 23,400 averages) to 77 s (SNR gain of 20 dB after 10,000 averages) resulted in no loss of detection accuracy. Reducing the measurement duration to approximately 30 s (SNR gain of 17.9 dB after 3900 averages) resulted in a degradation in the accuracy of only 1°. Further investigation revealed that a SNR of 20 dB (i.e., where the signal is two orders of magnitude larger than the noise) resulted in no loss of detection accuracy, while a SNR of 10 dB (i.e., where the signal is only one order of magnitude larger than the noise) resulted in an additional error of 2–3°.

### 4.7. Orientation Detection Accuracy and Sensor Orientation

The accuracy of electrode orientation detection depended on the orientation of the MEG sensors. The horizontal and symmetrical electrode configurations produced a magnetic field that mainly used a tangential field component at the sensor surface, marked as arrows in Figure 14d. However, each MEG sensor measured the normal field component, marked as arrows in Figure 14c, resulting in the measurement of an attenuated magnetic field, with the attenuation ranging from a factor of 3 (4.7 dB) to 62 (18 dB) depending on the angle between the sensor orientation and the direction of the magnetic field between approximately 80° and 110°, as shown in Figure 14e. On average, a SNR gain of approximately 8.1 dB was obtained, resulting in a dramatic reduction in measurement and an averaging time of less than 10 s.

### 4.8. Orientation Detection Accuracy and Localization Error

The accuracy of the electrode orientation detection depended on the localization error of the electrode. We assumed that the electrode was in the correct position, so the maximum GoF value of 1 was shifted to the correct orientation position (i.e., 99°). The change in GoF value and accuracy in detecting electrode orientation as a function of electrode displacement in all three Cartesian directions, from −15 mm to 15 mm in 1 mm increments, are provided in Figure 14f,g. Both the GoF value and the inaccuracy in the electrode orientation detection depended on the direction in which the electrode had been moved. Moving the electrode 1 mm in any direction had no negative effect on the accuracy of the electrode orientation detection, but still resulted in a slight decrease in the GoF value. Moving the electrode in the *z*-direction had the least impact on accuracy (even a localization error of 2–3 mm did not result in inaccuracies). A localization error of 2–3 mm, as occurs in MEG measurements, resulted in a decrease in accuracy of 1–2° in electrode orientation detection.

## 5. Discussion

In this study, we presented a radiation-free method for determining the position and orientation of a directional DBS electrode using a series of short SQUID-MEG measurements with different predefined electrode configurations. The potential of MEG recordings for such an application has already been demonstrated in our previous work [20,22]. The aim of this study was to develop a simple, realistic, and patient-friendly measurement method that can be used for clinical practice. The current step was necessary before measurements could be made with real DBS patients. A head–torso phantom with realistic dimensions was constructed to mimic a patient undergoing DBS therapy. The position and implantation angle of the electrode in the phantom were chosen to be as realistic as possible, and pre- and postoperative imaging were performed. Current state-of-the-art CT imaging techniques were applied to determine the position and rotational orientation of the electrode in the phantom, based on visual inspections of specific CT artifact patterns as the gold standard. These results were used to validate our MEG-based MaDoPO method. MEG measurements on the phantom were performed with different electrode configurations to find the sequence of measurements that achieves clinically reliable accuracy with the smallest amount of electrode contacts and the smallest possible number of recordings. Omnidirectional or directional configurations with clinically used stimulation parameters were applied for electrode localization and for electrode orientation detection, respectively.

The postoperative detection of electrode positions is essential for several aspects, such as to verify the implanted electrode position, to evaluate therapeutic and adverse effects of DBS, and to facilitate and guide stimulation programming [4]. Segmented contacts of a directional electrode may allow the stimulation field to be generated in a desired direction, so this technology also requires knowledge of the precise electrode orientation in the brain to fully realize its benefits. In addition, it is important to perform control localization and orientation detection when clinical doubts arise as to whether the stimulation is optimally adjusted for the patient, which requires CT imaging and/or fluoroscopy each time. The existing neuroimaging techniques have their own limitations and disadvantages, which were briefly discussed in the introduction. The major drawback is that all currently available techniques expose patients to ionizing radiation, and, in the case of orientation detection with X-ray or CT, none of the imaging techniques are currently able to resolve the 180° ambiguity in artifact symmetry without further measurements [16,32]. Therefore, possible imaging modalities such as EEG and MEG offer alternatives that eliminate these drawbacks, which are completely harmless and safe for the patient and do not involve an ambiguity problem. EEG has already been investigated for the feasibility of electrode localization, but insufficient accuracy for such problems has been reported [17].

In the method presented in this study, the magnetic field generated by the stimulation current is measured using MEG sensors and used to localize the electrode and detect its orientation. The accuracy in localization depended on the number of measurements with different non-directional electrode configurations. The DBS electrode in the phantom could be localized with an accuracy of approximately 2.25 mm, considering three specific measurements with bipolar non-directional configurations on each of the three levels of the electrode with a measurement duration of 50 s each. The accuracy could be improved to 2.02 mm when all six possible measurements with bipolar non-directional electrode configurations were considered. Three or six measurements per minute would result in a total pure measurement time of three to six minutes, i.e., a recording duration which can easily be tolerated by patients. The obtained inaccuracies can be attributed to the spatial resolution of the MEG system, since the sum of several inaccuracies in the preparation steps for the MEG measurements already caused a total error of 1.4 mm. This had the largest effect on the localization accuracy and indicated that the localization accuracy can be significantly improved if the spatial resolution of the MEG system is also improved. The accuracy in orientation detection depended on the number and combination of measurements with different directional electrode configurations. The orientation of the electrode was determined with an accuracy of 6° when three 77 s measurements were performed with the horizontal electrode configuration (i.e, where a segmented contact is activated against a neighboring segment). This error was reduced to 4° by using three additional measurements with the symmetrical electrode configuration (i.e., where two segmented contacts are activated against the third segment). Six measurements of one and a half minutes each would result in a pure measurement time of nine minutes in total, which would also be tolerable for the patient. The combination of the horizontal and symmetrical electrode configurations produced the best results compared to the combination options with the diagonal electrode configuration (i.e., where the electrode tip is activated against a single segment at the next higher level). Investigations revealed that both types of configurations complemented each other in terms of the direction of current flow generated by stimulation in the phantom, so finer sampling by the IPG’s MICC technology could further improve the accuracy in orientation detection. However, the accuracy was mainly limited by the spatial resolution of the MEG system, suggesting that a significant improvement in electrode orientation detection could be achieved if the measurement accuracy were improved.

Considering a postoperative localization accuracy of 2–3 mm and an orientation detection accuracy of 4–5°, according to our clinicians, these accuracies are acceptable for such DBS problems when gaining benefits of a radiation-free method. So, the presented method for detecting the position and orientation of a directional DBS electrode using magnetic field measurements continues to appear promising. Although the accuracies achieved in our study cannot compete with currently existing methods, the method we used is radiation-free and has the potential to be significantly improved with flexible head-casts for current SQUID-MEG devices [33] or with emerging MEG measurement systems, e.g., with OPM-MEG systems using optically pumped magnetometers (OPM) [23,24,34]. Recent technological developments have resulted in cap-shaped or on-scalp MEG sensor arrays that can move with the patients’ head and adapt to the individual head shape. This could greatly improve the spatial resolution of the system (which limited our accuracy) by eliminating the need to estimate the position of the head or phantom in the MEG system using attached HPI coils. This method could become a breakthrough in solving such DBS problems if it achieves high detection precision, and it could be the postoperative modality of choice to enable the full potential of individualized programming of directional DBS systems. Another possible improvement of detection accuracy results from a higher resolution discretization of the localization area, which was set to 1 mm for computational time reasons. If the electrode is not directly on a defined grid point, the localization error is at least the distance between the nearest grid point and the location of the electrode. Increasing the resolution can increase the detection accuracy, but drastically increases the computational complexity. Further investigation in this study has shown that the cap-shaped MEG system must have the following characteristics: (1) suitable magnetic field sensors that have the sensitivity and bandwidth required for this application [21]; (2) at least 40 and 60 sensors with concentrated distribution of sensors near the electrode for electrode localization and electrode orientation detection, respectively; (3) alignment of the sensors to the expected direction of the magnetic field (tangential component), with the direction of the magnetic field rotated 90° between directional and non-directional stimulation; (4) a pure measurement duration for a single recording of 50 and 77 s for localization and orientation detection, respectively; and (5) a minimum of three measurements with non-directional electrode configuration and a minimum of six measurements with directional configuration to achieve decent accuracy, although additional measurements may be considered to improve accuracy.

The presented method has significant limitations. We are aware that our measurements were performed under ideal conditions, and that the obtained accuracies in electrode localization and electrode orientation detection may decrease in real patients. In further research, the presented method needs to prove its practicability and clinical applicability in real data from DBS patients. Such data will be more challenging, because, on the one hand, biological artifacts (heart, eyes, brain activity, and motion) interfere with the measurements and, on the other hand, the heterogeneous and anisotropic tissue properties of the brain reduce the accuracy of the required electromagnetic model. However, in contrast, we know that the artifact produced by DBS stimulation is much larger than other artifacts with a significant increase in DBS amplitudes by using on-scalp MEG due to the tangential sensor orientation and the smaller distance between the sensors and the electrode. Since the current only flows near the electrode due to the bipolar electrode configuration, accurate modeling near the electrode and simplified modeling outside should also suffice. This needs to be clarified in future studies. Another disadvantage of this method is the time needed to localize the electrode and to detect the electrode’s orientation. The time mainly depends on the duration of the simulation to generate modeled data (expected magnetic field values at the sensor surface) for the localization algorithm. The simulations in our case, where the electrode was moved in steps of 1 mm within a defined region (29 × 29 × 29 mm), took approximately two weeks of computation time, so a finer resolution of the model or a larger region would drastically increase the computation time and thus the time to determine electrode position and orientation. Therefore, this method cannot currently be used for real time applications, e.g., during surgery, but only post-operatively, where time is basically irrelevant.

## 6. Conclusions

In this study, we presented an improved, realistic, patient-friendly, and radiation-free method for the magnetic detection of the position and orientation (MaDoPO) of a directional DBS electrode using conventional MEG. For this purpose, the magnetic field around a head–torso phantom with an integrated DBS system was measured under different non-directional and directional electrode configurations. Three specific measurements with bipolar non-directional stimulation over each level of the electrode resulted in a localization accuracy of approximately 2 mm. Six specific measurements with bipolar directional stimulation around the electrode resulted in an orientation detection accuracy of 4°. If this can be confirmed in human applications, it would be more than sufficient for clinical use. However, the use of emerging cap-shaped or on-scalp MEG systems, in which the spatial resolution of the system is improved, would significantly increase the accuracy in detection.

## Figures and Tables

**Table 1:** Performed MEG measurements.

Electrode Localization		Electrode Orientation Detection					
**Vertical Configuration**		**Diagonal Config.**		**Horizontal Config.**		**Symmetrical Config.**	
**No.**	**Contacts:** **(−) vs. (+)**	**No.**	**Contacts:** **(−) vs. (+)**	**No.**	**Contacts:** **(−) vs. (+)**	**No.**	**Contacts:** **(−) vs. (+)**
#01	C1 vs. C234	#07	C1 vs. C2	#13	C2 vs. C3	#19	C23 vs. C4
#02	C234 vs. C567	#08	C1 vs. C3	#14	C3 vs. C4	#20	C34 vs. C2
#03	C567 vs. C8	#09	C1 vs. C4	#15	C4 vs. C2	#21	C42 vs. C3
#04	C1 vs. C567	#10	C5 vs. C8	#16	C5 vs. C6	#22	C56 vs. C7
#05	C234 vs. C8	#11	C6 vs. C8	#17	C6 vs. C7	#23	C67 vs. C5
#06	C1 vs. C8	#12	C7 vs. C8	#18	C7 vs. C5	#24	C75 vs. C6

## Data Availability

Not applicable.
